# Safety of injective immunotherapy with monomeric carbamylated allergoid given to pediatric patients with allergic rhinitis with/without asthma due to house dust mite

**DOI:** 10.1186/1939-4551-8-S1-A20

**Published:** 2015-04-08

**Authors:** Enrico Compalati, Antonio Rinciani

**Affiliations:** 1 Allergy and Respiratory Diseases Clinic. University of Genoa, Italy; 2 Private Ambulatory of Pediatrics, Gela (Caltanissetta), Italy

## Background

The safety and efficacy of Monomeric Carbamylated Allergoid in injections have been previously reported in adult patients. The aim of this case-series was to explore its safety in a paediatric populations. Monomeric Carbamylated Allergoids are chemically modified extracts characterized by hypoallergenic activity due to allergen lysines substitution and preserved structural conformation and size of the native allergen.

## Methods

A total of 37 children (21 males, 16 females), mean age 8.06 years, were treated with Monomeric Allergoid in injections for house dust mite respiratory allergy (persistent rhinitis with/without mild asthma), and were followed up for a period of at least 12 months. After a build-up phase of 4 weeks (0.1 /0.2 /0.3 /0.5 mL per week), the treatment continued with monthly injection of 0.5mL. A standard questionnaire was used to collect data on local and systemic adverse reactions (ARs). The severity was graded as low (no need for treatment or dose adjustment), moderate (interference with activities/need for drugs/SCIT discontinuation), and severe (hospitalization/emergency care). A parental satisfaction on the clinical outcome (based on symptoms and drug consumption) was also collected (excellent, good, low, very low).

## Results

Only one patient interrupted the treatment after 6 months because he suffered from recurrent febrile upper respiratory infections. There were no serious ARs. Only at first administration we observed: 2 pain at the arm, 1 episode of headache, 2 of diffuse itching, 1 case of mild dyspnoea with no treatment interruptions, no hospitalizations and no dose-correlation. Parents’ satisfaction was judged excellent by 51%, good by 43.5%, low by 5.5% of the patients' parents.

## Conclusions

The safety of immunotherapy with Monomeric Allergoid through an injective route appears confirmed also in paediatric patients. High patients’ acceptance and satisfaction have been observed.

